# The discrepancy between preoperative cervical sagittal vertical axis and T1 slope predisposes inferior clinical outcomes in patients with cervical spondylotic myelopathy after cervical laminoplasty

**DOI:** 10.3389/fsurg.2022.1003757

**Published:** 2022-08-26

**Authors:** Dong-Fan Wang, Wei-Guo Zhu, Wei Wang, Xiang-Yu Li, Chao Kong, Cheng-Xin Liu, Bin Shi, Shi-Bao Lu

**Affiliations:** ^1^ Department of Orthopedics, Xuanwu Hospital, Capital Medical University, Beijing, China; ^2^ National Clinical Research Center for Geriatric Diseases, Xuanwu Hospital, Capital Medical University, Beijing, China

**Keywords:** cervical sagittal vertical axis, t1 slope, cervical laminoplasty, clinical outcomes, sagittal alignment

## Abstract

**Objective:**

Cervical sagittal parameters have been widely used to predict clinical outcomes in patients with cervical spondylotic myelopathy (CSM). This study aims to coin a novel cervical sagittal parameter defined as the ratio of cervical sagittal vertical axis to T1 slope (CSVA/T1S) and to investigate the correlation between CSVA/T1S and postoperative HRQOL after laminoplasty.

**Methods:**

A total of 102 CSM patients treated with cervical laminoplasty from our database were retrospectively reviewed. All patients were followed up for >12 months. Radiological parameters were measured using lateral cervical radiographs, including occiput-C2 lordosis (OC2), cervical lordosis (CL), CSVA, and T1S. Clinical parameters included the Japanese Orthopedic Association (JOA) score, neck disability index (NDI), and JOA recovery rate. Patients were grouped by preoperative T1S, T1S-CL, and CSVA/T1S value, respectively. Clinical and radiological outcomes were compared between the groups.

**Results:**

Patients with high CSVA/T1S had greater OC2 and CSVA but lower CL than those in the low CSVA/T1S group pre-and postoperatively. With respect to HRQOL results, the final NDI was 12.46 ± 9.11% in the low CSVA/T1S group, which was significantly lower than that in the high CSVA/T1S group (17.68 ± 8.81%, *P* = 0.040). Moreover, only CSVA/T1S was detected to be significantly correlated with final NDI (*r* = 0.310, *P* = 0.027). No significant correlation was found between clinical results and other cervical sagittal parameters, including T1S, CSVA, and T1S-CL.

**Conclusions:**

Preoperative CSVA/T1S was correlated with postoperative NDI in patients with CSM after cervical laminoplasty. Patients with low preoperative CSVA/T1S achieved better neurological function improvement after cervical laminoplasty. Cervical laminoplasty could be an appropriate choice for patients with lower preoperative CSVA/T1S.

## Introduction

Cervical spondylotic myelopathy (CSM) is a common cervical degenerative disorder with an incidence of about 4.04 per 100,000 person-years (1, 2), which is the leading cause of spinal cord dysfunction, including upper extremity numbness, weakness, decreased manual dexterity, and gait disturbance (3). Surgical intervention is usually indicated to halt neurological function deterioration and improve living quality for patients unresponsive to conservative treatment. Surgeries through anterior and posterior approaches are both important methods for the treatment of CSM. Compared with the anterior procedure, the posterior surgery is particularly well adapted to multi-level stenosis with spinal cord injury (4). Cervical laminoplasty, as the primary technique of posterior procedure, allows for direct posterior decompression in patients with myelopathy secondary to congenital cervical stenosis or hypertrophy of ligament flava and also affords indirect anterior decompression in patients with multiple disk herniations or ossification of the posterior longitudinal ligament (OPLL) (5).

However, cervical laminoplasty has its major defects that impair the cervical posterior muscle-ligament complex, which would lead to loss of cervical lordosis and then the cervical sagittal imbalance (5). It has been well documented that the cervical sagittal alignment, which could be reflected by cervical lordosis (CL), T1 slope (T1S), cervical sagittal axis (CSVA), the ratio of CL to T1S (CL/T1S), and T1S minus CL mismatch (T1S-CL), is correlated with patients' health-related quality of life (HRQOL) (6, 7). Among all the cervical parameters, CSVA was believed to be the primary factor influencing patients' self-reported outcomes such as neck pain and neurological symptoms before and after laminoplasty. In the *post hoc* analysis of a prospective and multicenter study, Smith et al. found that the preoperative modified Japanese Orthopedic Association (JOA) scores were correlated with CSVA in 56 patients with CSM (8). Kato et al. reported that the improvement in SF-36 physical component summary was significantly lower in patients with CSVA ≥ 35 mm after laminoplasty and concluded that CSVA could lead to poor postoperative HRQOL results and axial neck pain (9). Xu et al. also discovered that preoperative CSVA was significantly related to the neurological outcome after laminoplasty in their retrospective OPLL case study (10).

Therefore, figuring out the risk factors of cervical sagittal decompensation and suboptimal surgical outcome after cervical laminoplasty could serve as a significant reference for clinical practice. Like the decrease of sacral slope (SS) playing an essential role in compensating for global sagittal imbalance, T1S represents the compensatory capacity of the upper thoracic for cervical sagittal malalignment (11). We speculate that the preoperative relationship between CSVA and T1S, represented by the ratio of CSVA to T1S (CSVA/T1S), could reflect the possibility of spontaneous compensation of cervical sagittal malalignment and predict the clinical outcome after laminoplasty. We conduct the present study with the following aims: (1) to coin a novel cervical sagittal parameter CSVA/T1S and (2) to investigate the correlation between CSVA/T1S and postoperative HRQOL after laminoplasty.

## Materials and methods

### Patient population

After being approved by the Ethics Committee of Capital Medical University Xuanwu Hospital (approval number: 2018014), a retrospective review of patients who underwent cervical laminoplasty between March 2018 and February 2021 was performed. Patients with cervical myelopathy secondary to OPLL, congenital cervical stenosis, or multilevel cervical disk herniation were included in this study (5). Thereinto, those with the following manifestations were recommended for surgical treatment: (1) rapid progression of clinical signs and symptoms, (2) presence of the signs of myelopathy with or without radiculopathy for six months or longer, (3) compression ratio (canal diameter/vertebra diameter) approaching 0.4, and (4) with neutral to lordotic cervical sagittal alignment (12). Patients who met the inclusion criteria below were enrolled: (1) age >18 years, (2) complete preoperative and postoperative radiographic data, (3) followed up for at least 12 months. Patients with spinal tumors, tuberculosis, trauma, a history of spinal surgery or non-horizontal gaze when taking lateral cervical x-rays were excluded. A horizontal gaze was defined as −10° ≤ chin-brow to vertical angle ≤10° (13). A total of 102 patients were finally included in this study.

### Operative procedure

All the surgeries were performed based on the Hirabayashi method by the same spinal surgeon (14). Patients were positioned cranially 15–20° up in a prone position. The Mayfield skull clamp was used for immobilizing the head position. The incision was made on the posterior midline of the cervical skin. The spinous process, lamina, and bilateral lateral mass were exposed. Part of the spinous processes was removed using a rongeur for bone grafting on the hinge side. The paraspinal muscle of C2, especially the semispinalis, was preserved. A high-speed drill was used to create gutters on the bilateral laminae at the border of the laminae and facets. The lamina of the side with more significant clinical symptoms was completely cut and used as the open side; the other side of the lamina was partially cut with the ventral cortex preserved to form the hinge side. A thin-bladed Kerrison rongeur was used to remove ligamentum flava at the cranial and caudal ends of the intended laminar expansion to facilitate opening the lamina. Suitable Centerpiece laminoplasty plates (Medtronic Sofamor Danek) were respectively fixed on the lateral masses and laminae. Spacer height was dependent on the degree of canal stenosis. C-arm fluoroscopy was used to confirm the position of the plates. Additional bleeding can be stopped with the use of bipolar coagulation forceps and thrombinated gelfoam. A negative pressure drainage tube was indwelt on the open side. All patients were told to wear a collar for 4–6 weeks postoperatively. Postoperative rehabilitation was done as early as possible.

### Radiological parameters

**Figure 1:**
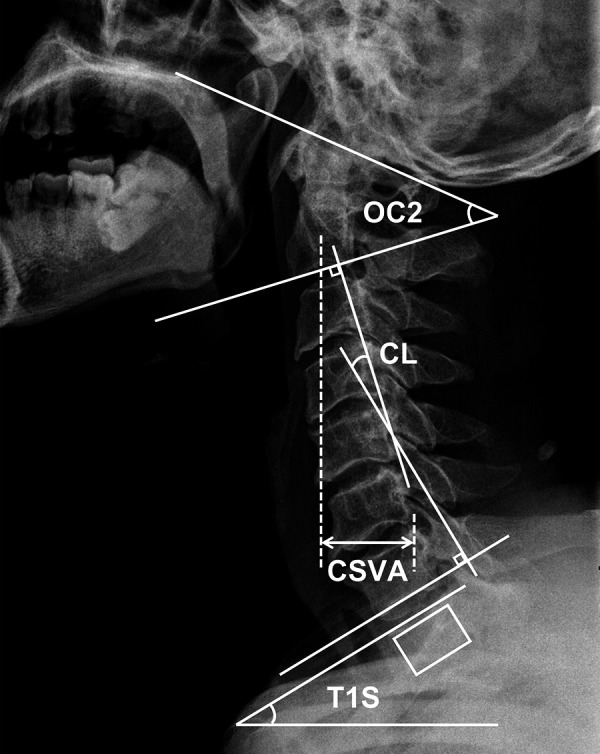
Measurements of cervical sagittal parameters utilized in this study.

A standing neutral lateral radiograph of the cervical spine was obtained with patients facing forward and in horizontal gaze before surgery and at the last follow-up. The following radiological parameters were measured: occiput-C2 lordosis (OC2, the angle between the McGregor line and the inferior endplate of the C2), CL (the angle between the inferior endplate of C2 and the inferior endplate of C7), T1S (the angle between a horizontal line and the superior endplate of T1), CSVA (the distance from the posterior, superior corner of C7 to the plumbline from the centroid of C2). To patients with invisible T1S on the cervical radiography, the value of superior C7 slope was utilized to substitute for T1S (15). All parameters were measured and calculated by 2 spine surgeons who were not involved in the program. For investigating the correlation between preoperative cervical sagittal parameters and HRQOL indicators after laminoplasty, all patients were grouped according to the median of preoperative T1S, T1S-CL, and CSVA/T1S, respectively. Figure 1 illustrated the cervical sagittal parameters measured in this study.

### Clinical parameters

The Japanese Orthopedic Association (JOA) score and neck disability index (NDI) were used to postoperative HRQOL (16, 17). The JOA recovery rate, calculated as (postoperative JOA score − preoperative JOA score)/(full score − preoperative JOA score) × 100%, was used to evaluate the improvement of cervical neurological function. A JOA recovery rate of 100% indicated being cured; >60% indicated significantly effective; 25%–60% indicated effective; <25% indicated ineffective. An NDI < 10% indicated no disability; 10%–30% indicated mild disability; 30%–50% indicated moderate disability; 50%–70% indicated severe disability; >70% indicated complete disability. Preoperative data were extracted from the medical charts. Postoperative data were collected from outpatient follow-up.

### Statistical analysis

All data were analyzed using SPSS Statistics (version 26.0, IBM Corp., Armonk, NY, USA). Data are presented as means ± standard deviations. Continuous variables were compared between groups using the independent-samples *t*-test, Mann-Whitney *U* test, and paired-sample *t*-test. The chi-square test was used to compare composition ratios. The correlations among the parameters were analyzed with Pearson correlation coefficient. Statistical significance was set at a level of *P* < 0.05.

## Results

### Baseline data of the whole cohort

A total of 102 patients were included in this study. They were 56 males and 46 females with an average age of 64.69 ± 9.73 years. The mean follow-up period was 17.88 ± 6.43 months. In terms of cervical sagittal parameters, OC2 angle was significantly increased from 23.34 ± 6.87° to 27.53 ± 7.21° (*P *< 0.001) whereas CL was significantly decreased from 13.99 ± 8.23° to 10.12 ± 7.78° (*P *< 0.001) after laminoplasty. As to clinical parameters, JOA score and NDI were both obviously improved. There were no significant differences between T1S and CSVA before and after surgery (Table 1).

**Table 1:** Comparison of cervical sagittal parameters and patient-reported outcome indicators pre- and postoperatively.

Parameters	Preoperative (*n* = 102)	Final follow-up (*n* = 102)	*P*
OC2 (°)	23.34 ± 6.87	27.53 ± 7.21	0.000**
CL (°)	13.99 ± 8.23	10.12 ± 7.78	0.000**
T1S (°)	24.52 ± 6.37	23.33 ± 6.86	0.167
CSVA (mm)	23.11 ± 11.97	24.65 ± 12.12	0.465
JOA score	12.01 ± 1.23	14.61 ± 1.18	0.000**
NDI (%)	29.54 ± 17.23	15.02 ± 9.26	0.000**

OC2, occiput-C2 lordosis; CL, cervical lordosis; T1S, T1 slope; CSVA, cervical sagittal vertical axis; JOA, Japanese orthopedic association; NDI, neck disability index.

** *P* < 0.01.

### Comparisons of cervical sagittal parameters and HRQOL outcomes between low T1S group and high T1S group

As shown in Table 2, the high T1S group had greater CL than the low T1S group pre-and postoperatively. Patients with high T1S tended to match high CSVA, though the data did not vary significantly between groups. Moreover, OC2 was significantly increased, while CL was decreased in both groups after surgery. Concerning the clinical parameters, no significant difference was found in terms of the JOA score, JOA recovery rate, and NDI between the two groups.

**Table 2:** Comparison of cervical sagittal parameters and HRQOL indicators between the low T1S group and the high T1S group.

Parameters	Low T1S (*n* = 51)	High T1S (*n* = 51)	*P*
Age (years)	63.92 ± 8.28	66.56 ± 10.43	0.207
Follow-up (months)	17.46 ± 6.55	18.32 ± 6.40	0.638
Operation segments			0.602
C3–C6	8	10	
C4–C7	13	9	
C3–C7	30	32	
OC2 (°)			
Pre-op	25.12 ± 6.78	24.11 ± 7.18	0.610
Final	27.82 ± 6.84***	27.43 ± 8.24***	0.853
CL (°)			
Pre-op	10.60 ± 7.73	17.54 ± 8.11	0.003**
Final	7.00 ± 7.78***	13.73 ± 7.69***	0.003**
T1S (°)			
Pre-op	19.47 ± 3.73	29.21 ± 3.85	0.000**
Final	19.05 ± 5.74	27.79 ± 5.12	0.000**
CSVA (mm)			
Pre-op	20.04 ± 9.30	26.59 ± 13.90	0.053
Final	21.21 ± 10.83	27.62 ± 13.65	0.069
JOA score			
Pre-op	12.15 ± 1.83	11.84 ± 1.95	0.556
Final	14.62 ± 1.30	14.60 ± 1.08	0.964
NDI (%)			
Pre-op	29.15 ± 15.94	31.76 ± 20.98	0.619
Final	15.77 ± 8.78	13.20 ± 9.56	0.171
JOA recovery rate (%)	51.25 ± 23.66	50.28 ± 20.39	0.876

OC2, occiput-C2 lordosis; CL, cervical lordosis; T1S, T1 slope; CSVA, cervical sagittal vertical axis; JOA, Japanese orthopedic association; NDI, neck disability index.

** *P* < 0.01.

*** There is a significant difference compared with preoperative parameter (*P *< 0.05).

### Comparisons of cervical sagittal parameters and HRQOL outcomes between low T1S-CL group and high T1S-CL group

Patients with high T1S-CL corresponding to greater OC2 and CSVA but lower CL than those with low T1S-CL. No significant difference was showed in T1S between groups. OC2 was increased and CL was decreased from the preoperative measurements through the final follow-up in both groups. JOA score, NDI, and JOA recovery rate were similar between the two groups (Table 3).

**Table 3:** Comparison of cervical sagittal parameters and HRQOL indicators between the low T1S-CL group and the high T1S-CL group.

Parameters	Low T1S-CL (*n* = 51)	High T1S-CL (*n* = 51)	*P*
Age (years)	63.08 ± 10.40	66.36 ± 8.87	0.232
Follow-up (months)	19.62 ± 7.13	17.08 ± 5.15	0.058
Operation segments			0.567
C3–C6	11	7	
C4–C7	10	12	
C3–C7	30	32	
OC2 (°)			
Pre-op	22.61 ± 6.75	26.72 ± 6.59	0.033*
Final	25.26 ± 7.88***	30.09 ± 6.30***	0.020*
CL (°)			
Pre-op	19.01 ± 7.59	8.80 ± 6.18	0.000**
Final	13.86 ± 8.76***	6.60 ± 6.20***	0.001**
T1S (°)			
Pre-op	22.92 ± 6.14	25.62 ± 6.04	0.121
Final	23.78 ± 7.44	23.77 ± 5.85	0.995
CSVA (mm)			
Pre-op	18.45 ± 12.06	30.49 ± 10.08	0.000**
Final	16.51 ± 9.85	30.27 ± 10.21	0.000**
T1S-CL (°)			
Pre-op	3.91 ± 4.98	16.82 ± 5.03	0.000**
Final	9.93 ± 5.37***	17.18 ± 7.34	0.000**
JOA score			
Pre-op	12.42 ± 1.92	11.56 ± 1.76	0.101
Final	14.77 ± 1.21	14.04 ± 1.15	0.326
NDI (%)			
Pre-op	32.08 ± 17.68	28.72 ± 19.42	0.521
Final	14.23 ± 10.53	15.84 ± 7.85	0.538
JOA recovery rate (%)	51.76 ± 23.65	49.55 ± 20.36	0.747

OC2, occiput-C2 lordosis; CL, cervical lordosis; T1S, T1 slope; CSVA, cervical sagittal vertical axis; JOA, Japanese orthopedic association; NDI, neck disability index.

* *P* < 0.05.

** *P* < 0.01.

*** There is a significant difference compared with preoperative parameter (*P *< 0.05).

### Comparisons of cervical sagittal parameters and HRQOL outcomes between low CSVA/T1S group and high CSVA/T1S group.

Table 4 detailed the comparison of sagittal and clinical parameters between patients with different CSVA/T1S. Compared with the low CSVA/T1S group, OC2 and CSVA were significantly greater and CL was significantly lower in the high CSVA/T1S group. Both CSVA and CSVA/T1S were increased in the Low CSVA/T1S group after surgery (*P* = 0.028), while they were not obviously changed in the high CSVA/T1S group. With respect to HRQOL results, the final NDI was 12.46 ± 9.11% in the low CSVA/T1S group, which was significantly lower than that in the High CSVA/T1S group (17.68 ± 8.81%, *P* = 0.040).

**Table 4:** Comparison of cervical sagittal parameters and HRQOL indicators between the low CSVA/T1S group and the high CSVA/T1S group.

Parameters	Low CSVA/T1S (*n* = 51)	High CSVA/T1S (*n* = 51)	*P*
Age (years)	63.88 ± 8.76	65.60 ± 9.99	0.334
Follow-up (months)	18.58 ± 6.40	17.16 ± 6.50	0.437
Operation segments			0.266
C3–C6	7	11	
C4–C7	9	13	
C3–C7	35	27	
OC2 (°)			
Pre-op	21.69 ± 6.66	27.68 ± 5.89	0.001**
Final	24.67 ± 7.71***	30.71 ± 5.94***	0.003**
CL (°)			
Pre-op	17.82 ± 7.89	10.03 ± 7.52	0.001**
Final	13.03 ± 8.50***	7.46 ± 7.39***	0.016*
T1S (°)			
Pre-op	24.64 ± 4.83	23.83 ± 7.42	0.645
Final	24.30 ± 7.03	22.33 ± 6.67	0.310
CSVA (mm)			
Pre-op	15.48 ± 9.06	31.34 ± 9.33	0.000**
Final	20.05 ± 12.72***	30.83 ± 10.98	0.011*
CSVA/T1S			
Pre-op	0.63 ± 0.36	1.35 ± 0.29	0.000**
Final	0.86 ± 0.54***	1.30 ± 0.38	0.001**
JOA score			
Pre-op	12.04 ± 1.89	11.96 ± 1.90	0.883
Final	14.77 ± 1.24	14.44 ± 1.12	0.326
NDI (%)			
Pre-op	27.15 ± 19.62	33.84 ± 16.84	0.199
Final	12.46 ± 9.11	17.68 ± 8.81	0.040*
JOA recovery rate (%)	51.30 ± 24.56	50.23 ± 19.24	0.863

OC2, occiput-C2 lordosis; CL, cervical lordosis; T1S, T1 slope; CSVA, cervical sagittal vertical axis; JOA, Japanese orthopedic association; NDI, neck disability index.

* *P* < 0.05.

** *P* < 0.01.

*** There is a significant difference compared with preoperative parameter (*P *< 0.05).

### Correlation analysis

Table 5 summarized the correlations between cervical sagittal parameters and clinical results. Only CSVA/T1S was detected to be correlated with final NDI (*r* = 0.310, *P* = 0.027). No significant correlation was found between clinical results and T1S, CSVA, or T1S-CL.

**Table 5:** Correlations between T1S, CSVA, T1S-CL, CSVA/T1S, final JOA score, JOA recovery rate, final NDI.

		Final JOA score	JOA recovery rate	Final NDI
CSVA/T1S	Pearson coefficients	−0.096	−0.111	0.310
	*P*	0.501	0.439	0.027*
T1S-CL	Pearson coefficients	−0.003	0.153	−0.068
	*P*	0.984	0.284	0.635
CSVA	Pearson coefficients	−0.168	0.024	0.264
	*P*	0.237	0.870	0.062
T1S	Pearson coefficients	−0.038	0.170	−0.133
	*P*	0.790	0.233	0.351

CSVA, cervical sagittal vertical axis; T1S, T1 slope; CL, cervical lordosis; JOA, Japanese orthopedic association; NDI, neck disability index.

* *P* < 0.05.

## Discussion

Posterior cervical laminoplasty has been proven to be a classical surgical technique for CSM, which can generate an indirect decompression by allowing the spinal cord to migrate dorsally. However, this technique always has a major defect damaging the posterior muscular-ligament complex, which often leads to the loss of cervical sagittal lordosis and a tendency of tilting forward (manifest as CSVA increasing) (18). In a retrospective study including 108 patients with CSM, Pan et al. found that the CL decreased from 13.9° to 10.7°, and the CSVA increased from 21 mm to 25 mm after cervical laminoplasty (19). Lin et al. also reported a significant increase in CSVA from 20.8 mm to 25.7 mm and a decrease of C3–C7 curvature from 11.6° to 7.8° in 37 patients with CSM treated by open-door laminoplasty (20). In the present study, 71.5% (73 / 102) of patients underwent a decrease in CL after cervical laminoplasty. The mean CL angle was decreased from 13.99 ± 8.23° to 10.12 ± 7.78° (*P* < 0.001). Cervical sagittal malalignment has been demonstrated to have an impact on surgical outcomes, including neck pain and neurological symptoms after laminoplasty (21).

T1S plays an essential role in the evaluation of cervical sagittal alignment after laminoplasty. Kim et al. prospectively analyzed the effect of T1S on kyphotic alignment change after cervical laminoplasty in 51 patients with CSM and found that those with high T1S had more postoperative kyphotic alignment changes (22). Zhang et al. further demonstrated that preoperative T1S had a positive correlation with postoperative loss of cervical lordosis (LCL) after laminoplasty (23). However, patients with high T1S and those with low T1S underwent a similar extent of CL decreasing and final HRQOL indicators in this study (Tables 2, 5). Consistent with our results, Cho et al. illustrated that the aggravation of cervical sagittal alignment and changes in HRQOL indicators were not associated with preoperative T1S in patients with CSM who underwent cervical laminoplasty (24). Furthermore, T1S in both groups did not differ significantly before and after surgery (Table 2). If CL decreasing could not be compensated by T1S, the cervical spine would present anterior malalignment in the sagittal plane, which always has negative effects on surgical outcomes, especially the HRQOL scores (6, 25).

Like the relationship between SS and LL, the harmony between cervical alignment and cervicothoracic alignment is the objective that spinal surgeons are trying to obtain. Previous studies have proven that the preoperative mismatch between CL and T1S would induce inferior outcomes before and after laminoplasty. In patients with OPLL who underwent laminoplasty, Kim et al. found that lower T1S-CL was significantly associated with more postoperative CL decreasing (26). Li et al.'s results showed that the JOA recovery rate after cervical laminoplasty in 78 CSM patients with extreme CL/T1S was significantly lower than that in patients with fair CL/T1S, which indicated that patients with high CL/T1S ratio would have inferior HRQOL results (27). Similarly, Rao et al. found that patients with T1S-CL mismatching are more likely to have postoperative kyphotic alignment changes, cervical sagittal imbalance, and unsatisfied self-reported outcomes (28). In the current study, the cervical alignment of the high T1S-CL group showed a relatively anterior malalignment. The CL decreased significantly after laminoplasty in both groups. However, there were no significant differences in clinical parameters between groups according to our research, and the T1S-CL was not correlated with the final JOA score, JOA recovery rate, and NDI (Tables 3, 5).

Compared with CL, CSVA would have more relevance with HRQOL scores. We speculate that the match between CSVA and T1S could be more efficient in predicting the clinical outcome after laminoplasty. In this study, we put forward a novel cervical sagittal parameter CSVA/T1S, which could represent the capacity of T1S to compensate for cervical sagittal malalignment. Interestingly, we found that high preoperative CSVA/T1S was correlated with worse NDI at the final follow-up in patients with CSM who underwent cervical laminoplasty, while CSVA was not (Table 5). In addition, results indicated that T1S had little difference, but CSVA was quite various between the high and low CSVA/T1S groups (15.48 ± 9.06 vs. 31.34 ± 9.33, *P* < 0.001), which meant the change of CSVA/T1S was mainly affected by CSVA (Table 4). Therefore, the effect of CSVA on clinical outcomes after cervical laminoplasty was amplified by matching with T1S. Moreover, though CSVA in the low CSVA/T1S group increased after surgery, it was still significantly lower than that in the high CSVA/T1S group and closer to the normal range as reported by Hardacker et al. in asymptomatic patients of 16.8 ± 11.2 mm (29). High CSVA/T1S refers to the condition that cervical sagittal malalignment might be beyond the compensatory power of T1S. Then the residual malalignment would increase the spinal cord intramedullary pressures and the tension in the neck muscle, which contribute to unsatisfied neurological function recovery and inferior postoperative HRQOL results (30). For those with Low CSVA/T1S, the upper thoracic spine had sufficient potential to halt anterior cervical malalignment, which would be helpful for maintaining better sagittal balance after laminoplasty. Thus, the final NDI result in the low CSVA/T1S group was superior.

The present study used CSVA/T1S to predict the HRQOL after cervical laminoplasty for the first time. According to our research, patients with high preoperative CSVA/T1S had higher NDI at the final follow-up after cervical laminoplasty. Inversely, patients with low preoperative CSVA/T1S corresponded to better neurological function recovery from cervical laminoplasty. In summary, results indicated that patients with low preoperative CSVA/T1S might be better candidates for cervical laminoplasty. There are still several limitations to our study that need to be considered. Firstly, our research is retrospective, only the data contained in the medical records can be analyzed. Secondly, the sample size was relatively limited and from a single center. Thirdly, the thoracolumbar and the lower limb parameters, which might influence the cervical spine alignment, were not included. Further studies will be necessary to assess the effect of CSVA/T1S on clinical outcome predicting and surgical decision-making. Despite these, our findings could still serve as a reference for spinal surgeons when making a surgical plan for patients with CSM.

### Patient presentation

**Figure 2:**
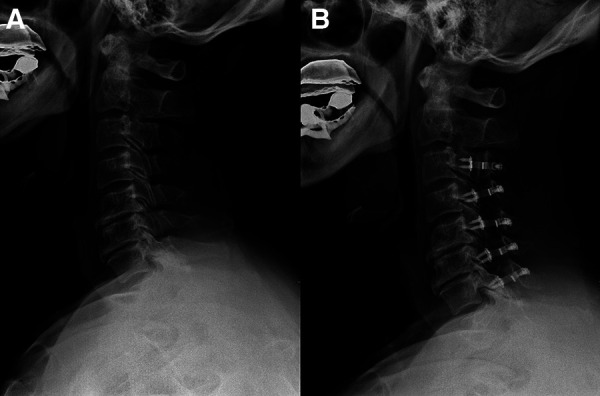
A 65-year-old male patient treated with C3–C7 laminoplasty. (**A**) Preoperative lateral cervical radiograph (CSVA = 15.4 mm, T1S = 23.4°). (**B**) Lateral cervical radiograph at final follow-up (17 months after surgery, CSVA = 17.5 mm).

**Patient 1 (Low CSVA/T1S, C3–7 laminoplasty**; Figure 2**)**: A 65-year-old male with a 17-month follow-up. The preoperative CSVA/T1S value was 0.66 (CSVA: 15.4 mm, T1S: 23.4°). Preoperative JOA score and NDI were 12 and 28.9%, respectively. At the final follow-up, the change of CSVA was +2.1 mm. JOA score increased from 12 to 16, while NDI decreased from 28.9% to 12%. The JOA recovery rate was 80%.

**Figure 3:**
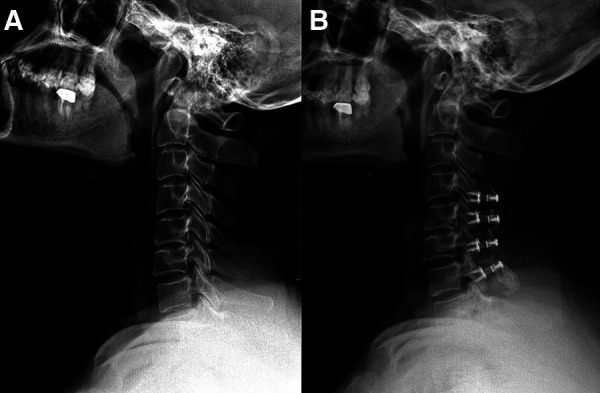
A 60-year-old male patient treated with C4–C7 laminoplasty. (**A**) Preoperative lateral cervical radiograph (CSVA = 9.7 mm, T1S = 20.7°). (**B**) Lateral cervical radiograph at final follow-up (19 months after surgery, CSVA = 11 mm).

**Patient 2 (Low CSVA/T1S, C4–7 laminoplasty**; Figure 3): A 60-year-old male with a 19-month follow-up. The preoperative CSVA/T1S value was 0.47 (CSVA: 9.7 mm, T1S: 20.7°). Preoperative JOA score and NDI were 11 and 26%, respectively. At the final follow-up, the change of CSVA was +1.3 mm. JOA score increased from 11 to 15, while NDI decreased from 26% to 14%. The JOA recovery rate was 67%.

**Figure 4:**
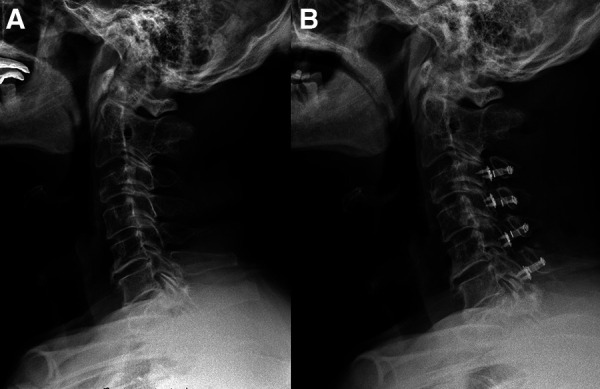
A 63-year-old male patient treated with C4–C7 laminoplasty. (**A**) Preoperative lateral cervical radiograph (CSVA = 29.5 mm, T1S = 21.8°). (**B**) Lateral cervical radiograph at final follow-up (18 months after surgery, CSVA = 30.9 mm).

**Patient 3 (High CSVA/T1S, C4–7 laminoplasty**; Figure 4**)**: A 63-year-old male with an 18-month follow-up. The preoperative CSVA/T1S value was 1.35 (CSVA: 29.5 mm, T1S: 21.8°). Preoperative JOA score and NDI were 10 and 30%, respectively. At the final follow-up, the change of CSVA was +1.4 mm. JOA score increased from 10 to 15, while NDI decreased from 30% to 18%. The JOA recovery rate was 71%.

**Figure 5:**
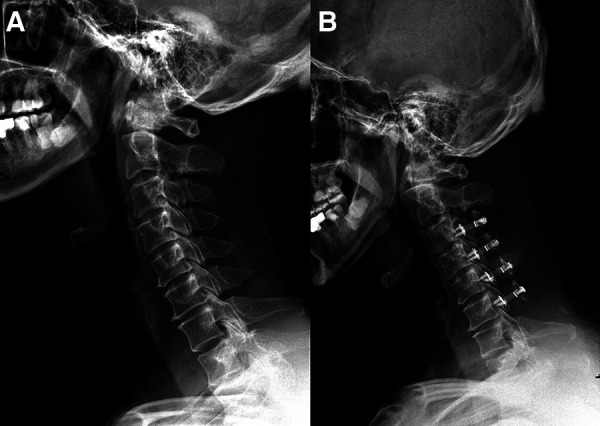
A 58-year-old male patient treated with C3–C6 laminoplasty. (**A**) Preoperative lateral cervical radiograph (CSVA = 37.6 mm, T1S = 28°). (**B**) Lateral cervical radiograph at final follow-up (17 months after surgery, CSVA = 41.2 mm).

**Patient 4 (High CSVA/T1S, C3–6 laminoplasty**; Figure 5): A 58-year-old male with a 17-month follow-up. The preoperative CSVA/T1S value was 1.34 (CSVA: 37.6 mm, T1S: 28°). Preoperative JOA score and NDI were 10 and 28%, respectively. At the final follow-up, the change of CSVA was +3.6 mm. JOA score increased from 10 to 14, while NDI decreased from 28% to 17.7%. The JOA recovery rate was 57%.

## Conclusion

Preoperative CSVA/T1S was significantly correlated with postoperative NDI in patients with CSM after cervical laminoplasty. Patients with low preoperative CSVA/T1S achieved better neurological function improvement after cervical laminoplasty. Cervical laminoplasty could be an appropriate choice for patients with lower preoperative CSVA/T1S.

## Data Availability

The original contributions presented in the study are included in the article/Supplementary Material, further inquiries can be directed to the corresponding author/s.

## References

[1] Bernhardt M Hynes RA Blume HW White AA. Cervical spondylotic myelopathy. J Bone Joint Surg Am. (1993) 75(1):119–28. 10.2106/00004623-199301000-00016 8419381

[2] Wu JC Ko CC Yen YS Huang WC Chen YC Liu L Epidemiology of cervical spondylotic myelopathy and its risk of causing spinal cord injury: a national cohort study. Neurosurg Focus. (2013) 35(1):E10. 10.3171/2013.4.FOCUS13122 23815246

[3] Emery SE. Cervical spondylotic myelopathy: diagnosis and treatment. J Am Acad Orthop Surg. (2001) 9(6):376–88. 10.5435/00124635-200111000-00003 11767723

[4] Chiba K Ogawa Y Ishii K Takaishi H Nakamura M Maruiwa H Long-term results of expansive open-door laminoplasty for cervical myelopathy–average 14-year follow-up study. Spine. (2006) 31(26):2998–3005. 10.1097/01.brs.0000250307.78987.6b 17172996

[5] Cho SK Kim JS Overley SC Merrill RK. Cervical laminoplasty: indications, surgical considerations, and clinical outcomes. J Am Acad Orthop Surg. (2018);26(7):e142–e52. 10.5435/JAAOS-D-16-00242 29521698

[6] Hyun SJ Han S Kim KJ Jahng TA Kim HJ. Assessment of T1 slope minus cervical lordosis and C2-7 sagittal vertical axis criteria of a cervical spine deformity classification system using long-term follow-up data after multilevel posterior cervical fusion surgery. Oper Neurosurg. (2019) 16(1):20–6. 10.1093/ons/opy055 29617850

[7] Stegen S van Gastel N Carmeliet G. Bringing new life to damaged bone: the importance of angiogenesis in bone repair and regeneration. Bone. (2015) 70:19–27. 10.1016/j.bone.2014.09.017 25263520

[8] Smith JS Lafage V Ryan DJ Shaffrey CI Schwab FJ Patel AA Association of myelopathy scores with cervical sagittal balance and normalized spinal cord volume: analysis of 56 preoperative cases from the AOSpine North America myelopathy study. Spine. (2013) 38(22 Suppl 1):S161–70. 10.1097/BRS.0b013e3182a7eb9e 23963001

[9] Kato M Namikawa T Matsumura A Konishi S Nakamura H. Effect of cervical sagittal balance on laminoplasty in patients with cervical myelopathy. Global Spine J. (2017) 7(2):154–61. 10.1177/2192568217694011 28507885 PMC5415157

[10] Xu C Zhang Y Dong M Wu H Yu W Tian Y The relationship between preoperative cervical sagittal balance and clinical outcome of laminoplasty treated cervical ossification of the posterior longitudinal ligament patients. Spine J. (2020) 20(9):1422–9. 10.1016/j.spinee.2020.05.542 32474225

[11] Lee DH Ha JK Chung JH Hwang CJ Lee CS Cho JH. A retrospective study to reveal the effect of surgical correction of cervical kyphosis on thoraco-lumbo-pelvic sagittal alignment. Eur Spine J. (2016) 25(7):2286–93. 10.1007/s00586-016-4392-9 26810979

[12] Petraglia AL Srinivasan V Coriddi M Whitbeck MG Maxwell JT Silberstein HJ. Cervical laminoplasty as a management option for patients with cervical spondylotic myelopathy: a series of 40 patients. Neurosurgery. (2010) 67(2):272–7. 10.1227/01.NEU.0000371981.83022.B1 20644412

[13] Scheer JK Tang JA Smith JS Acosta FL Jr Protopsaltis TS Blondel B Cervical spine alignment, sagittal deformity, and clinical implications: a review. J Neurosurg Spine. (2013) 19(2):141–59. 10.3171/2013.4.SPINE12838 23768023

[14] Hirabayashi K Watanabe K Wakano K Suzuki N Satomi K Ishii Y. Expansive open-door laminoplasty for cervical spinal stenotic myelopathy. Spine(1983) 8(7):693–9. 10.1097/00007632-198310000-00003 6420895

[15] Tamai K Buser Z Paholpak P Sessumpun K Nakamura H Wang JC. Can C7 slope substitute the T1 slope?: an analysis using cervical radiographs and kinematic MRIs. Spine. (2018) 43(7):520–5. 10.1097/BRS.0000000000002371 28767624

[16] Kato S Oshima Y Oka H Chikuda H Takeshita Y Miyoshi K Comparison of the Japanese Orthopaedic Association (JOA) score and modified JOA (mJOA) score for the assessment of cervical myelopathy: a multicenter observational study. PLoS One. (2015) 10(4):e0123022. 10.1371/journal.pone.0123022 25837285 PMC4383381

[17] Howell ER. The association between neck pain, the Neck Disability Index and cervical ranges of motion: a narrative review. J Can Chiropr Assoc. (2011) 55(3):211–21. 21886283 PMC3154067

[18] Aita I Wadano Y Yabuki T. Curvature and range of motion of the cervical spine after laminaplasty. J Bone Joint Surg Am. (2000) 82(12):1743–8. 10.2106/00004623-200012000-00008 11130648

[19] Pan Y Ma X Feng H Chen C Qin Z Huang Y. Effect of posterior cervical expansive open-door laminoplasty on cervical sagittal balance. Eur Spine J. (2020) 29(11):2831–7. 10.1007/s00586-020-06563-9 32776264

[20] Lin S Zhou F Sun Y Chen Z Zhang F Pan S. The severity of operative invasion to the posterior muscular-ligament complex influences cervical sagittal balance after open-door laminoplasty. Eur Spine J. (2015) 24(1):127–35. 10.1007/s00586-014-3605-3 25307698

[21] Zhang X Gao Y Gao K Yu Z Lv D Ma H Factors associated with postoperative axial symptom after expansive open-door laminoplasty: retrospective study using multivariable analysis. Eur Spine J. (2020) 29(11):2838–44. 10.1007/s00586-020-06494-5 32524286

[22] Kim TH Lee SY Kim YC Park MS Kim SW. T1 slope as a predictor of kyphotic alignment change after laminoplasty in patients with cervical myelopathy. Spine. (2013) 38(16):E992–7. 10.1097/BRS.0b013e3182972e1b 23609205

[23] Zhang JT Li JQ Niu RJ Liu Z Tong T Shen Y. Predictors of cervical lordosis loss after laminoplasty in patients with cervical spondylotic myelopathy. Eur Spine J. (2017) 26(4):1205–10. 10.1007/s00586-017-4971-4 28168336

[24] Cho JH Ha JK Kim DG Song KY Kim YT Hwang CJ Does preoperative T1 slope affect radiological and functional outcomes after cervical laminoplasty? Spine. (2014) 39(26):E1575–81. 10.1097/BRS.0000000000000614 25271514

[25] Knott PT Mardjetko SM Techy F. The use of the T1 sagittal angle in predicting overall sagittal balance of the spine. Spine J. (2010) 10(11):994–8. 10.1016/j.spinee.2010.08.031 20970739

[26] Kim B Yoon DH Ha Y Yi S Shin DA Lee CK Relationship between T1 slope and loss of lordosis after laminoplasty in patients with cervical ossification of the posterior longitudinal ligament. Spine J. (2016) 16(2):219–25. 10.1016/j.spinee.2015.10.042 26523967

[27] Li XY Kong C Sun XY Guo MC Ding JZ Yang YM Influence of the ratio of C2-C7 cobb angle to T1 slope on cervical alignment after laminoplasty. World Neurosurg. (2019) 124:e659–66. 10.1016/j.wneu.2018.12.181 30654159

[28] Rao H Huang Y Lan Z Xu Z Li G Xu W. Does preoperative T1 slope and cervical lordosis mismatching affect surgical outcomes after laminoplasty in patients with cervical spondylotic myelopathy? World Neurosurg. (2019) 130:e687–e93. 10.1016/j.wneu.2019.06.193 31279919

[29] Hardacker JW Shuford RF Capicotto PN Pryor PW. Radiographic standing cervical segmental alignment in adult volunteers without neck symptoms. Spine. (1997) 22(13):1472–80; discussion 80. 10.1097/00007632-199707010-00009 9231966

[30] Tang JA Scheer JK Smith JS Deviren V Bess S Hart RA The impact of standing regional cervical sagittal alignment on outcomes in posterior cervical fusion surgery. Neurosurgery. (2015) 76(Suppl 1):S14–21; discussion S. 10.1227/01.neu.0000462074.66077.2b 25692364

